# Green-Synthesization of Silver Nanoparticles Using Endophytic Bacteria Isolated from Garlic and Its Antifungal Activity against Wheat *Fusarium* Head Blight Pathogen *Fusarium graminearum*

**DOI:** 10.3390/nano10020219

**Published:** 2020-01-27

**Authors:** Ezzeldin Ibrahim, Muchen Zhang, Yang Zhang, Afsana Hossain, Wen Qiu, Yun Chen, Yanli Wang, Wenge Wu, Guochang Sun, Bin Li

**Affiliations:** 1 State Key Laboratory of Rice Biology and Ministry of Agriculture Key Lab of Molecular Biology of Crop Pathogens and Insects, Institute of Biotechnology, Zhejiang University, Hangzhou 310058, China; ezzelbehery8818@yahoo.com (E.I.); 11816060@zju.edu.cn (M.Z.); 0618151@zju.edu.cn (Y.Z.); afsana_07@yahoo.com (A.H.); wenwen20101010@163.com (W.Q.); chenyun0927@zju.edu.cn (Y.C.); 2 Department of Vegetable Diseases Research, Plant Pathology Research Institute, Agriculture Research Centre, Giza 12916, Egypt; 3 Department of Plant Pathology and Seed Science, Sylhet Agricultural University, Sylhet 3100, Bangladesh; 4 State Key Laboratory for Quality and Safety of Agro-products, Institute of Plant Protection and Microbiology, Zhejiang Academy of Agricultural Sciences, Hangzhou 310021, China; sungc01@sina.com; 5 Rice Research Institute, Anhui Academy of Agricultural Sciences, Hefei 230001, China

**Keywords:** *F. graminearum*, nanoparticles, SEM, FTIR, antifungal activity, mycotoxins

## Abstract

Nanoparticles are expected to play a vital role in the management of future plant diseases, and they are expected to provide an environmentally friendly alternative to traditional synthetic fungicides. In the present study, silver nanoparticles (AgNPs) were green synthesized through the mediation by using the endophytic bacterium *Pseudomonas poae* strain CO, which was isolated from garlic plants (*Allium sativum*). Following a confirmation analysis that used UV–Vis, we examined the in vitro antifungal activity of the biosynthesized AgNPs with the size of 19.8–44.9 nm, which showed strong inhibition in the mycelium growth, spore germination, the length of the germ tubes, and the mycotoxin production of the wheat *Fusarium* head blight pathogen *Fusarium graminearum*. Furthermore, the microscopic examination showed that the morphological of mycelia had deformities and collapsed when treated with AgNPs, causing DNA and proteins to leak outside cells. The biosynthesized AgNPs with strong antifungal activity were further characterized based on analyses of X-ray diffraction, transmission electron microscopy, scanning electron microscopy, EDS profiles, and Fourier transform infrared spectroscopy. Overall, the results from this study clearly indicate that the biosynthesized AgNPs may have a great potential in protecting wheat from fungal infection.

## 1. Introduction

*Fusarium* head blight caused by *Fusarium graminearum* is a great obstacle to wheat production in the world [1]. This fungal pathogen is also able to infect other cereal crops such as rice, barley, and maize [1,2,3]. In China, this pathogen has not only resulted in a heavy loss of more than 3.41 million tons in wheat during 2000–2018 [4,5], it has also been able to produce some mycotoxins in infected wheat grains that pose a serious threat to the safety and health of humans and animals [6,7,8]. Nowadays, chemical fungicides are used as the main strategy to protect wheat from this fungal infection. However, their long-time use has resulted in the increased fungicide resistance of the fungal strains [9]. In addition, concerns have been raised regarding the safety of fungicides to human health, the environment and the ecosystem [10]. Therefore, it is very necessary to develop a new alternate method to inhibit or kill this pathogen.

Various metals nanoparticles have been widely applied in agriculture due to their distinctive features such as antimicrobial activity, catalytic activity, magnetic and electrical properties [11,12]. Furthermore, several studies have demonstrated that metal nanoparticles have been well documented as fungicides [10,13]. For example, silver nanoparticles (AgNPs) have been shown to significantly inhibit the hyphal growth of a variety of foliar and soil-borne plant pathogen [14,15]. In addition, more and more attention has been paid to the biosynthesis of AgNPs by using eco-friendly materials, such as plant extracts and microorganisms, which are safer than traditional physical and chemical methods [16,17,18,19].

In this study, we biosynthesized AgNPs by using the endophytic bacterium *Pseudomonas poae* strain CO, and then we characterized the AgNPs with strong antifungal activity against *F. graminearum*. In addition, we also evaluated the antifungal mechanism of the biosynthesized AgNPs against *F. graminearum*. 

## 2. Materials and Methods 

### 2.1. Microorganisms Used in This Study

*F. graminearum* strain PH−1 [20] was kindly provided by Professor Yanni Yin in the Institute of Biotechnology, Zhejiang University, Hangzhou, China. The endophytic bacterium *P. poae* strain CO was isolated from fresh leaves of a garlic plant grown in Hangzhou, Zhejiang Province, China. This strain was identified through 16S rRNA gene sequence analysis.

### 2.2. Biosynthesis of AgNPs

In this study, the endophytic bacterium *P. poae* strain CO was used to mediate the biosynthesis of AgNPs, and this process was performed according to the method of Fouad et al. [21] with slight modification. In brief, the endophytic bacterium *P. poae* strain CO was inoculated in a nutrient broth (10 g of tryptone, 3 g of beef extract, 2.5 g of glucose, and 5 g of NaCl per liter; pH 7.0; all ingredients were purchased from Sangon Biotech, Shanghai, and then incubated at 30 ℃ at 200 rpm for 2 days. Ten milliliters of culture filtrates (CF) were mixed with 90 mL of 3 mM aqueous of silver nitrate (Cat. no. 10018461; Sinopharm, Shanghai, China) in a 250 mL Erlenmeyer flask, followed by shaking at 200 rpm, 30 ℃ for 4 days in darkness. A nutrient broth of the same volume was used as the control. The biosynthesis of AgNPs was noted by the change of the color from light yellow to dark brown, while the formation of AgNPs was assured by UV–visible spectrometry from 200 to 800 nm at a 1 nm resolution by using a Shimadzu UV-2550 spectrometer (Shimadzu, Kyoto, Japan). The pellets were collected by centrifuging at 10,000 g for 20 min and washing twice with double distilled water (ddH_2_O). The biosynthesized AgNPs were freeze-dried for future use.

### 2.3. Antifungal Activity of the Biosynthesized AgNPs

#### 2.3.1. Inhibition of AgNPs on Mycelium Growth

The inhibitory effect of AgNPs of 5, 10, 15 and 20 µg/mL on the mycelium growth of *F. graminearum* strain PH−1 was determined according to the method of Spence et al. [22] in both a potato dextrose agar (PDA) medium, which consisted of 200 g of potato infusion, 20 g of dextrose and 20 g of agar per liter (pH 7.0), and potato dextrose broth (PDB), which consisted of 200 g of potato infusion and 20 g of dextrose per liter (pH 7.0). The mycelium growth in the PDA medium was measured by inoculating a disk (5 mm in diameter) of 5-day-old fungus in the middle of a dish that contained the mixture of the PDA medium with different concentrations of AgNPs and then incubating at 27 ℃ for 5 days. The PDA medium without AgNPs was used as the control. The fungal growth in the PDA broth was determined by inoculating a disk (5 mm in diameter) of 5-day-old fungus in a PDA broth containing AgNPs of different concentrations and then measuring the dry weight of the mycelium after 5 days of incubation at 27 ℃. The PDB medium without AgNPs was used as the control. 

#### 2.3.2. Effect of AgNPs on Spore Germination and Length of Germ Tubes

The effect of AgNPs at 5, 10, 15 and 20 µg/mL on the spore germination and length of the germ tubes of *F. graminearum* strain PH−1 were determined according to the method of Chen et al. [23] with slight modifications. In brief, a 100 μL of spore suspension (1 × 10^6^ spores per mL) that were prepared as described by Wu et al. [24] were mixed with 100 μL of AgNPs in the tubes with final concentrations of 5, 10, 15 and 20 µg/mL. The tubes containing the same volume of spore suspensions and ddH_2_O was used as the control. Then, 50 μL of the mixture was transferred onto concave slides and incubated at 28 ℃ for 7 h in the dark. The spore germination rate and the length of germ tubes were recorded by using the light microscope. Each treatment had three replicates, and the experiment was repeated twice.

#### 2.3.3. Effect of AgNPs on Deoxynivalenol Production

The inhibition of the deoxynivalenol (DON) production of *F. graminearum* strain PH−1 was tested according to the method of Li et al. [25] with slight modifications. In brief, a 1 mL of spore suspension (1 × 10^6^ spores/mL) of *F. graminearum* strain PH−1 was inoculated with 100 mL of GYEP that contained the indicated concentrations of AgNPs. The Erlenmeyer flask was incubated at 28 ℃ and 175 rpm for one week. The supernatant was used to determine DON production by using DON Plate Kit ELISA (Shanghai Yijishiye, Zhenjiang, China), and the inhibition of DON was calculated. 

### 2.4. Antifungal Mechanism of the Biosynthesized AgNPs

#### 2.4.1. Effect of AgNPs on the Hyphal Morphology

The effect of AgNPs on the hyphal morphology of *F. graminearum* strain PH−1 was determined based on the method used by Gao et al. [26] with slight modifications. In brief, a mycelial disk (10 mm in diameter) was picked up from the PDA medium with and without AgNPs (10 µg/mL) and then observed by using both scanning electron microscopy (SEM; TM−1000, Hitachi, Japan) and transmission electron microscopy (TEM; JEM−1230, JEOL, Akishima, Japan).

#### 2.4.2. Effect of AgNPs on the Leakage of DNA and Proteins 

The effect of AgNPs on the leakage of DNA and proteins from *F. graminearum* strain PH−1 cells was determined by measuring the optical density (OD) of the spore supernatant at 260 and 280 nm, and this process was performed according to the method of Khalil et al. [16] with slight modifications. In brief, the spore suspension (1 × 10^6^ spores/mL) was prepared by incubating *F. graminearum* strain PH−1 at 28 ℃ for 24 h in the PDB medium, which was supplemented with four different concentrations of AgNPs. The PDB medium without AgNPs was used as a control. 

### 2.5. Characterization of the Biosynthesized AgNPs

The functional group of the biosynthesized AgNPs with antifungal activity was determined by Fourier transform infrared spectroscopy (FTIR), which was carried out as described by Hossain et al. [20]. In brief, 1 mg of freeze-dried AgNP powders were mixed with KBr (300 mg), and the FTIR was recorded in the spectral range of 500–4000 cm by using an AVATAR 370 FTIR spectrometer (Thermo Nicolet, MA, USA). Furthermore, the crystalline nature of the biosynthesized AgNPs was analyzed by X-ray diffraction (XRD), as described by Hossain et al. [20] by using an XPert PRO diffractometer (Holland) with a detector voltage of 45 kV and a current of 40 mA while using CuKo radiation. In addition, the morphology of the biosynthesized AgNPs was recorded through the observation of both TEM (JEM−1230, JEOL, Tokyo, Japan) and SEM (SEM, TM−1000, Hitachi, Japan), which were performed according to the method of [18]. The silver element of the AgNPs was confirmed with an energy dispersive spectrometer (EDS). 

### 2.6. Statistical Analysis

All experiments were done by using a completely randomized design, and the results are expressed as mean ± SD (standard deviation). Statistical analysis was performed by using the SPSS software package 16.0 version (SPSS Inc., Chicago, IL, USA). The variations between the groups were estimated by using the analysis of the different test. The results were statistically significant when the value was *p* < 0.05 or < 0.01.

## 3. Results and Discussion

### 3.1. Biosynthesis and Confirmation of AgNPs

**Figure 1:**
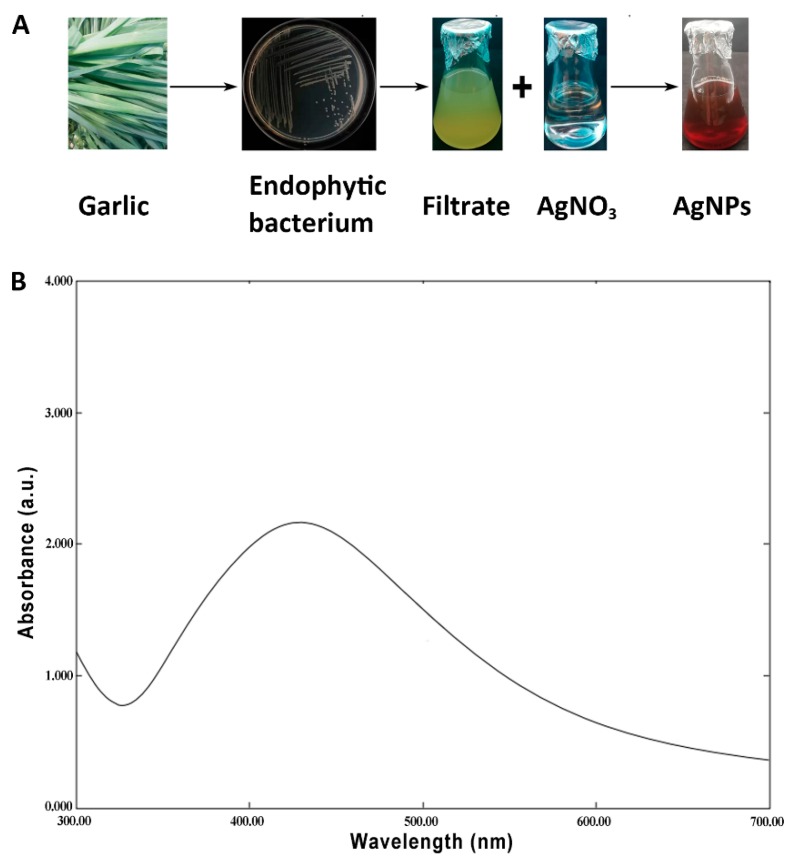
Confirmation of biosynthesized silver nanoparticles (AgNPs). (**A**) Color changes from light yellow to dark brown following the incubation of AgNO_3_ with the culture filtrates of the endophytic bacteria *Pseudomonas poae* strain CO isolated from garlic. (**B**) UV–Vis spectrum of AgNPs that were synthesized by the endophytic bacteria *P. poae* strain CO.

The procedure for the biosynthesis of AgNPs is shown in Figure 1. The synthesis of AgNPs was justified based on the result that the color changed from light yellow to dark brown following the incubation of AgNO_3_ with the CF of endophytic bacteria for four days, suggesting Ag^+^ reduction to Ag^0^ in the AgNO_3_ solution. In contrast, no change was observed in the color of the control sample. This result suggests that the CF of endophytic bacteria may play a key role in the biosynthesis of AgNPs. Furthermore, the formation of AgNPs was confirmed by the results of UV spectrometers, which showed a spectrum of surface plasmon resonance (SPR) at the 422 nm absorption band (Figure 1). Interestingly, the result of this study is consistent with the spectra contained in the literature regarding silver nanoparticles [20,22,27,28]. 

This study first reported that the endophytic bacterium *P. poae* that was isolated from garlic plants was able to mediate the biosynthesis of AgNPs. These bacteria have been used as a biological source in the biosynthesis of AgNPs, but the mechanism of bacteria for AgNPs biosynthesis is still unknown. Interestingly, a lot of previous reports have revealed the existence of various biomolecules that are produced by bacteria such as alkaloids, phenolic compounds, enzymes, co-enzymes, proteins, amino acids, polysaccharides, and vitamins, all of which have been able to act as effective agents for converting Ag^+^ to Ag^0^ [19,20,29,30]. Therefore, it could be inferred that these biomolecules that are produced by bacteria may play a key role in the mediation of AgNPs biosynthesis.

### 3.2. Antifungal Activity and Mechanism

#### 3.2.1. Inhibition of Mycelium Growth

**Figure 2:**
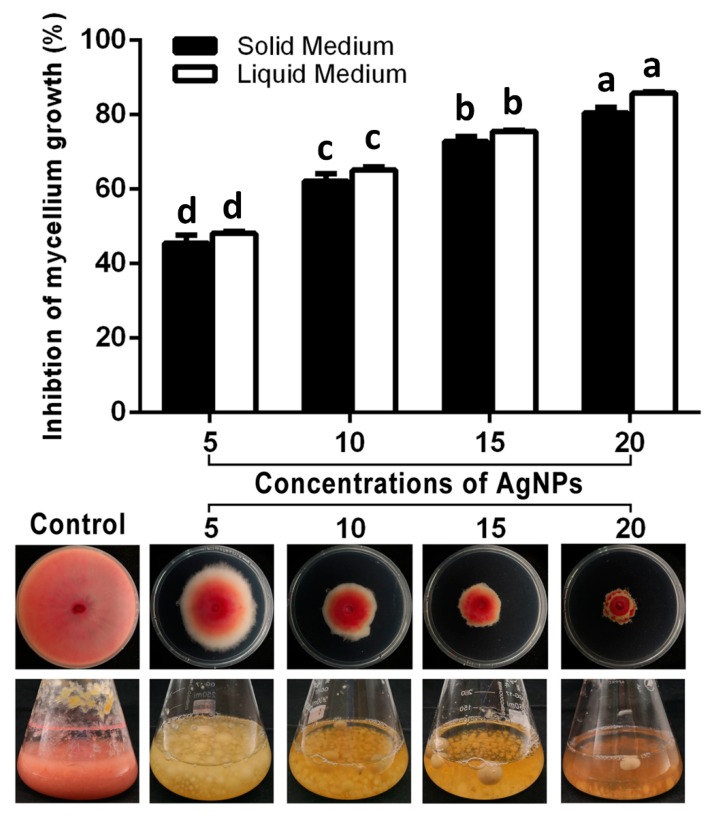
In vitro antifungal activity of AgNPs biosynthesis at different concentrations against *Fusarium graminearum* strain PH−1 in potato dextrose agar (PDA) and potato dextrose broth (PDB) mediums. Data are a mean value ± standard error of three replicates, and bars with the same letters are significantly different in LSD test (*p* < 0.05).

The results of this study showed that the mycelium growth of *F. graminearum* strain PH−1 in PDA and PDB mediums was strongly suppressed by AgNPs. regardless of the concentration, as shown in Figure 2. The inhibitory effect on the mycelium growth in the PDA and PDB mediums increased with the increase of the AgNP concentrations. Indeed, AgNPs at 5, 10, 15 and 20 μg/mL caused 45.56%, 62.22%, 72.78% and 80.56%, respectively, inhibitions in the mycelium growth in the PDA medium, and they caused 48.56%, 65.11%, 75.50% and 85.78%, respectively, inhibitions in the mycelium growth in the PDB medium. In agreement with results of this study, previous studies have indicated that AgNPs are able to be used as antifungal agents to suppress many fungal plant diseases [15,30,31,32,33,34,35].

#### 3.2.2. Damage of Cell Walls by Morphological Observation 

**Figure 3:**
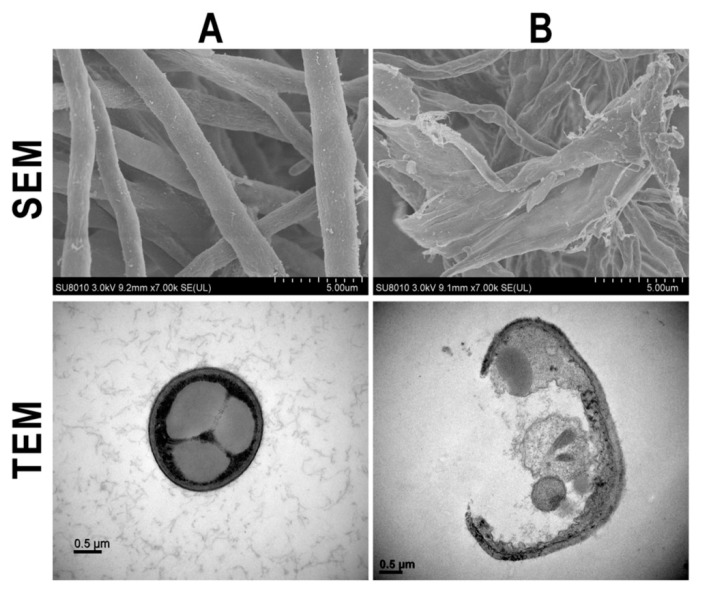
Microscopic images of SEM and TEM of *F. graminearum* strain PH−1 in the absence (**A**) and presence (**B**) of the synthesized AgNPs.

The fungal cell wall is a complex structure that plays a key role in determining cell shape. Furthermore, the cell wall is able to protect fungal cells from environmental stress, including changes in osmolality, temperature and pH [11,30,36]. The data from the use of SEM and TEM in this study indicated that cell wall remained intact with hyphae, showing a normal structural property of *F. graminearum* strain PH−1 in the absence of biosynthesized AgNPs. In contrast, the cell walls of *F. graminearum* strain PH−1 was severely damaged and the hyphae showed an abnormal structural property in the presence of the biosynthesized AgNPs (Figure 3). Similar effects of AgNPs have been reported on the cell walls of several other pathogenic fungi such as *Alternaria alternata*, *Botrytis cinera* and *Trichosporon asahii* [37].

#### 3.2.3. Damage of Cell Walls by Determination of Leakage DNA and Proteins

**Figure 4:**
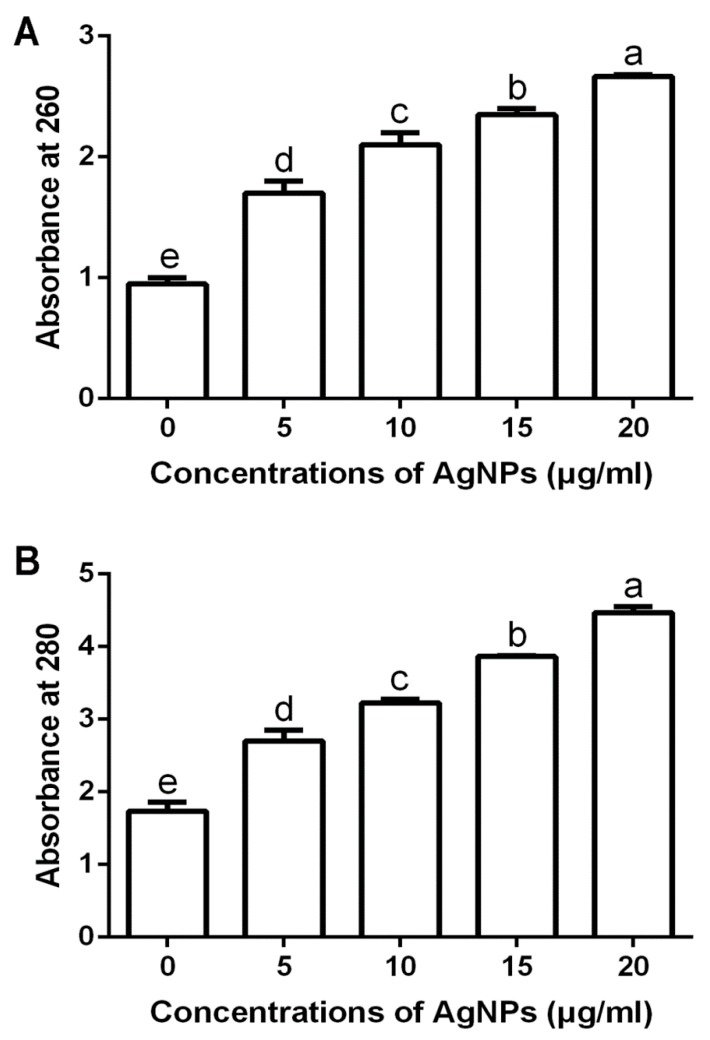
Biosynthesized AgNPs at different concentrations caused the leakage of DNA (**A**) and proteins (**B**) from *F. graminearum* strain PH−1 spore cells. Data are a mean value ± standard error of three replicates, and bars with the same letters are significantly different in LSD test (*p* < 0.05).

The results from this study indicated that the biosynthesized AgNPs of different concentrations caused the leakage of DNA and proteins from *F. graminearum* strain PH−1 spore cells. Furthermore, the leakage of DNA and proteins increased with the increase of the concentration of the biosynthesized AgNPs (Figure 4). Indeed, the OD260 and OD280 values of strain PH−1 spore cells were 0.95 and 1.74, respectively, in the absence of the biosynthesized AgNPs. However, in the presence of the biosynthesized AgNPs at 5, 10, 15 and 20 μg/mL, the OD260 values were 1.70, 2.10, 2.45 and 2.67, respectively (Figure 4A), while the OD280 values were 2.70, 3.23, 3.86 and 4.47, respectively (Figure 4B). In agreement with the results of this study, previous studies have indicated that the accumulation of AgNPs on the cell membrane is able to cause cell membrane damage, which results in leakage and the release of cellular contents, including DNA and proteins [16,30,38].

#### 3.2.4. Inhibition of Spore Germination and Germ Tube Growth

**Figure 5:**
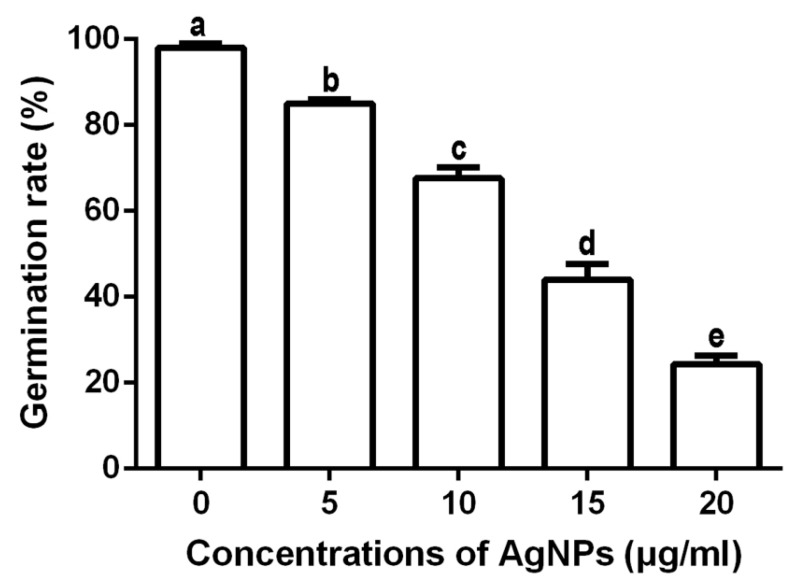
Effect of the synthesized AgNPs of different concentrations on spores’ germination rate of *F. graminearum* strain PH−1. The biosynthesized AgNPs at four different concentrations were able to effectively suppress the spore germination. Data are a mean value ± standard error of three replicates, and bars with the same letters are significantly different in LSD test (*p* < 0.05).

**Figure 6:**
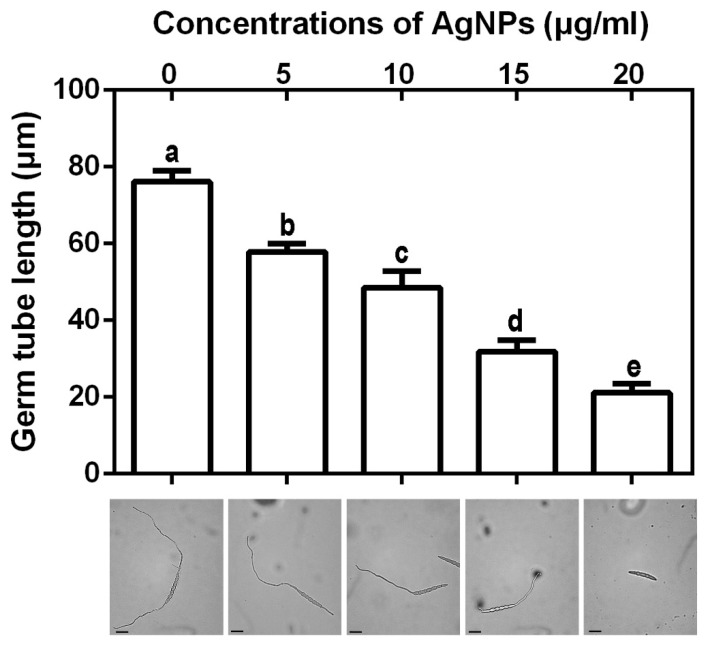
Effect of the synthesized AgNPs at four different concentrations on germ tube growth. The biosynthesized AgNPs were able to inhibit germ tube growth of *F. graminearum* strain PH−1. Data are a mean value ± standard error of three replicates, and bars with the same letters are significantly different in LSD test (*p* < 0.05).

The results of this study indicated that the biosynthesized AgNPs at four different concentrations were able to effectively suppress the spore germination and germ tube growth of *F. graminearum* strain PH−1. The inhibitory effect increased with the increase of the concentration of the biosynthesized AgNPs. Indeed, the spore germination rate was 98.00%, while the length of the germ tubes was 76.14 μm in absence of the biosynthesized AgNPs. In the presence of AgNPs at 5, 10, 15 and 20 μg/mL, the spore germination rates were 85.00%, 67.67%, 44.00% and 24.33%, respectively (Figure 5), and the germ tube lengths were 57.86, 48.57, 31.86, and 21.14 μm (Figure 6), respectively. It is well known that germinated spores have a key role in colonization and infection of pathogenic fungi to plants [24,39]. Therefore, the inhibition of spore germination will greatly reduce the infection risk of these fungal pathogens to wheat plants. AgNPs have been observed to have a significant effect on the length and pattern of germ tubes.

#### 3.2.5. Effect of AgNPs on DON Production

**Figure 7:**
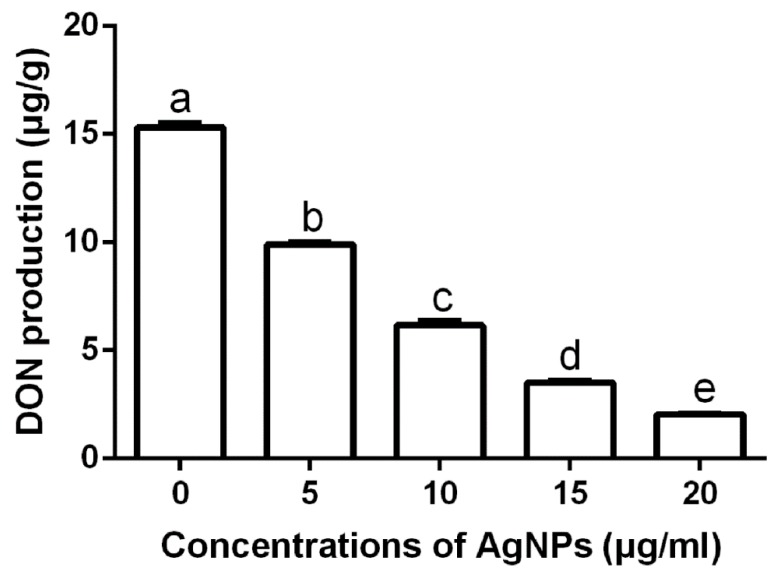
Effect of the synthesized AgNPs of different concentrations on deoxynivalenol (DON) production. The production of the DON of *F. graminearum* strain PH−1 was inhibited by the biosynthesized AgNPs in vitro. Data are a mean value ± standard error of three replicates, and bars with the same letters are significantly different in LSD test (*p* < 0.05).

DON is one of the most important mycotoxins that causes damage to human and animal health [40]. The results of this study showed that the DON production of *F. graminearum* strain PH−1 in the GYEP medium was strongly inhibited by the biosynthesized AgNPs at different concentrations. In the absence of the biosynthesized AgNPs, the DON production rate of strain PH−1 was 15.32 μg/g. In the presence of the biosynthesized AgNPs at 5, 10, 15, and 20 μg/mL, the DON production rates were 9.91, 6.22, 3.50 and 2.04 μg/g, respectively, (Figure 7). The result of this study suggest that biosynthesized AgNPs have a great potential in reducing DON contamination in cereals that are infected by *F. graminearum*. In agreement with the result of our study, biosynthesized AgNPs have been found to be effective in thwarting the production of the mycotoxins of *Aspergillus flavus* and *Aspergillus ochraceus* [16].

### 3.3. Characterization of the Biosynthesized AgNPs

**Figure 8:**
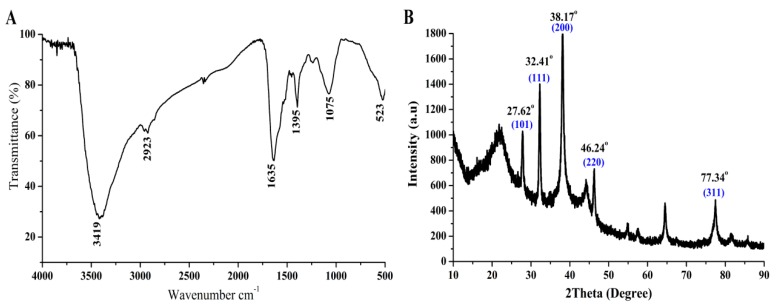
Characterization of the biosynthesized AgNPs after four days of incubation. (**A**) Fourier-transform infrared spectrum of biosynthesized AgNPs. (**B**) X-ray diffraction patterns of the biosynthesized AgNPs.

The biosynthesized AgNPs with strong antifungal activity were further characterized based on the analysis of FTIR, XRD, TEM, SEM and EDS in this study. The functional groups of the synthesized AgNPs were identified based on the FTIR analysis. Indeed, six peaks at 3419, 2923, 1635, 1395, 1075, and 523 cm^−1^ were observed in the FTIR spectra of biosynthesized AgNPs, as shown in Figure 8A. Based on previous publications, these peaks were able to be attributed to the corresponding functional groups. For example, the peak at 3419 cm^−1^ was assigned to the NH stretching of Amide A [41]. The peak at 2923 Cm^−1^ indicated the CH stretch of alkanes [42]. The peak at 1635 Cm^−1^ could be denoted by the carbonyl stretching vibration [43]. The peak at 1395 cm^−1^ indicated the O–C–H, C–C–H and C–O–H bending vibrational modes of the carbohydrates [44]. The peaks at 1075 and 523 cm^−1^ could be assigned to the (C–O) of an alkoxy group [45] and CH_2_ groups [46], respectively. 

In agreement with the finding of other studies [22,47], the results of this study suggest that the crystallization of bio-organic compounds may occur on the surface of biosynthesized AgNPs. Indeed, the crystalline nature of the biosynthesized AgNPs was determined based on the result of XRD analysis, which indicated that there were five emission peaks of 2θ = 27.62°, 32.41°, 38.17°, 46.24° and 77.34°, corresponding to the silver crystal planes (101), (111), (200), (220), and (311), respectively (Figure 8B).

**Figure 9:**
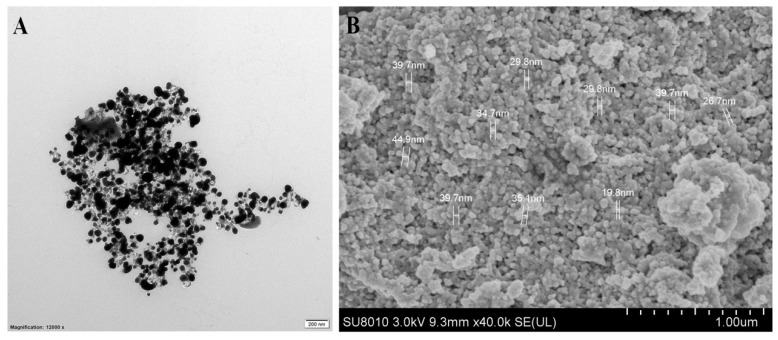
Size and morphology of silver nanoparticles (AgNPs) that were synthesized by reducing 3 mM AgNO_3_ by using culture filtrates of the endophytic bacterium *P. poae* strain CO. (**A**) transmission electron microscopy and (**B**) scanning electron microscopy.

**Figure 10:**
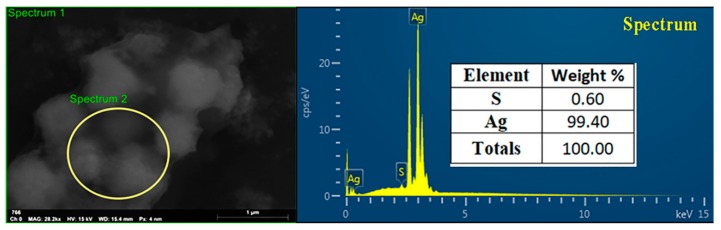
Energy dispersive spectrum showing the predominance of Ag and S elements in the AgNPs.

The structure and morphological shape of the biosynthesized AgNPs were determined by using both TEM and SEM observation. The results of this study indicated that the biosynthesized AgNPs were spherical and had a size of 19.8–44.9 nm (Figure 9). In general, the results are consistent with the data from a lot of previous reports [20,22,48]. Furthermore, the results of EDS showed that the peak elements of silver and sulfur were 99.40% and 0.60%, respectively, in the biosynthesized AgNPs (Figure 10). In addition, in agreement with the results obtained in previous studies [17,49], the peak of the silver ions was formed at 3 KeV, a fact that could be useful in reducing Ag^+^ to Ag^0^.

## 4. Conclusions 

To overcome the disadvantages caused by physical and chemical methods, biological methods are a safe alternative for the synthesis of AgNPs. In this study, the biosynthesis of AgNPs was first carried out by an endophytic bacterium that was isolated from garlic. The formation of AgNPs was confirmed by visible UV spectroscopy. In addition, the biosynthesized AgNPs showed strong antifungal activity against the *Fusarium* head blight pathogen *F. graminearum* strain PH−1, which may be at least partially attributed to their inhibition on spore germination, germ tube growth, mycotoxin production, and the damage of cell membrane. Furthermore, the biosynthesized AgNPs with strong antifungal activity were characterized by FTIR, XRD, SEM, TEM, and EDS analyses. In general, the result of this study indicate that the synthesized AgNPs have a great potential to protect wheat plants from fungal infection.
